# Spatiotemporal evolution of healthcare service capacity at township health centers in China

**DOI:** 10.3389/fpubh.2023.1229453

**Published:** 2023-12-08

**Authors:** Hong Chen, Liyang Zhao, Jin Yu

**Affiliations:** College of Public Administration and Law, Hunan Agricultural University, Changsha, Hunan, China

**Keywords:** Township health center, healthcare service capacity, unexpected output superefficiency SBM, exploratory spatiotemporal data analysis, quantile regression

## Abstract

**Introduction:**

This study analyzes the efficiency, spatiotemporal evolution, and influencing factors of provincial township health centers' healthcare service capacity in China.

**Method:**

It utilizes an unexpected output super-efficiency slacks-based measure (SBM) model, exploratory spatiotemporal data analysis methods, and a quantile regression model.

**Results:**

The results show that the healthcare service capacity of township health centers is better in provinces with a larger proportion of hierarchical diagnoses and treatments pilot projects in cities, and the regional efficiency trend is ordered central > eastern > western > northeastern. The healthcare service capacity of provincial township health centers mainly shows significant spatial correlation and a spatiotemporal distribution pattern of “high agglomeration, low differentiation.”

**Discussion:**

Rural population density and per capita GDP significantly improve the healthcare service capacity of township health centers, while local governments' healthcare and health expenditure increases the healthcare service capacity of township health centers in certain quantiles. The urbanization rate and per capita disposable income inhibit the improvement of the healthcare service capacity of township health centers in certain quantiles. The provinces should accelerate the promotion of hierarchical diagnoses and treatment pilot projects in cities and establish national cooperative development models to promote public health.

## 1 Introduction

The 19th National Congress of the Communist Party of China proposed the construction of a “Healthy China” by promoting the expansion of high-quality healthcare resources, achieving a balanced distribution, improving disease prevention and treatment at the grassroots level, and focusing on rural areas and communities. China's primary healthcare system is mainly composed of community health service centers and township health centers, with the former primarily serving urban residents and the latter mainly serving rural residents (1). With the implementation of hierarchical diagnoses and system pilot projects in cities and the continuous rapid growth of rural residents' healthcare needs with improved living standards, the healthcare service capacity of township health centers has significantly improved (2). According to the China Health Statistical Yearbook (3), as of 2021, China's rural township health centers had 2.84 beds per 1,000 people, 2.58 health technical personnel per 1,000 people, and 1.161 billion outpatient visits and inpatient admissions, with growth rates of 8.65, 6.02, and 2.37% per year, respectively, compared with the data from 2009. Both policies and data demonstrate that since the implementation of the New Healthcare Reform, the healthcare service capacity of China's township health centers has made remarkable achievements (4), is undergoing a transition from “scale expansion” to “quality improvement,” and is showing signs of advancing to a higher level. The reform strategy of “maintaining basic health services, strengthening primary healthcare, and establishing a mechanism” has been proposed since the New Healthcare Reform was implemented. Improving the primary healthcare service capacity is the best interpretation of “strengthening primary healthcare.” However, China still faces problems such as inadequate primary healthcare service capacity (5), low primary healthcare service efficiency (6), and an uneven distribution of primary healthcare resources (7). Therefore, this study aims to explore the spatiotemporal evolution characteristics, leaping laws, and driving factors of the healthcare service capacity of township health centers in each province. By analyzing and presenting solutions for these issues, the results may assist policymakers in formulating macro-control policies that are suitable for China's healthcare system reform.

## 2 Literature review

Scholars at home and abroad have provided different definitions of healthcare service capacity based on three perspectives: resource, hierarchical, and functional. From the resource perspective, most scholars believe that human (8), financial (9), and material resources (10) constitute healthcare service capacity. From the hierarchical perspective, He and Yu (11) defined the primary healthcare service capacity using three levels: macro, meso, and micro, and asserted that primary healthcare service capacity is the resource allocation and operation mechanism of the primary healthcare service system, with the competence to continuously and effectively meet the primary healthcare service needs of the people. From the functional perspective, basic healthcare service capacity refers to the healthcare service capacity that primary healthcare centers can provide to the general public that is affordable and can meet their health needs (12).

Regarding the measurement of primary healthcare service capacity, scholars at home and abroad have mainly conducted empirical analyses from the efficiency perspective, and have frequently used data envelopment analysis (DEA). For example, Samut and Cafri (13) used DEA to evaluate hospital efficiency in 29 member countries of the Organization for Economic Cooperation and Development (OECD), and used efficiency as the dependent variable to analyze the impact of environmental factors on hospital efficiency using a tobit regression model. Ahmed (14) also used DEA to evaluate healthcare centers' management performance and technical efficiency. Yang (15) argues that although DEA is commonly used, the efficiency value of the decision-making unit (DUM) can only be 1, while the unexpected output super-efficiency slacks-based measure (SBM) model can allow the efficiency value of the decision-making unit to exceed 1, which is more applicable when measuring efficiency.

In recent years, research on spatiotemporal evolution has gradually increased and has been widely used in fields such as efficiency analysis (16), industrial structures (17), and resource environments (18). Han et al. (19) states that differential structures exhibit dynamic changes, including spatial and temporal attributes. Exploratory spatiotemporal data analysis methods are mainstream methods that combine spatial and temporal attributes for empirical analysis. Tim (20) proposes that these methods can help explain complex spatiotemporal relationships and can play a key role in multiple disciplines. Li and Zeng (21) used exploratory spatiotemporal data analysis methods to explore the spatiotemporal evolution characteristics of public health levels in Chinese cities, and found that these methods could more accurately depict healthcare centers' spatial distribution characteristics.

However, some deficiencies remain in research on the primary healthcare service capacity. First, regarding its efficiency analysis, most scholars have used the traditional DEA method. Although some scholars have used the non-expected output super efficiency SBM model to analyze the efficiency of healthcare services in China, it is only applied to the macro level measurement of healthcare service capacity efficiency and cannot explain the regional differences in grassroots healthcare service capacity (15, 22). Second, regarding spatiotemporal evolution, some scholars have used exploratory spatiotemporal data analysis methods (23); however, there is relatively little research on the driving factors of the spatiotemporal leap of primary healthcare service capacity using this method.

Therefore, this study uses the unexpected output super-efficiency SBM model to analyze the efficiency of the primary healthcare service capacity, with relevant data on township health centers in Chinese provinces from 2009 to 2021. It applies exploratory spatiotemporal data analysis methods to explore the heterogeneity and transition patterns of the primary healthcare service capacity, and uses quantile regression to analyze the driving factors of the spatiotemporal transition of the primary healthcare service capacity in each province. The results may provide a reference for policymakers to develop targeted policies that can enhance the healthcare service capacity of township health centers at the provincial level.

## 3 Materials and methods

### 3.1 Non-desirable output-super efficiency SBM model

In 2001, Tone (24) proposed the non-desirable output SBM (Super Efficiency SBM) model. This model differs from the traditional DEA model in that it addresses the issue of not considering slack variables due to the radial and angular problems between inputs and outputs. Tone later discovered that the model could not distinguish the results when all decision-making units were equal to 1. Therefore, he improved the SBM model and introduced the concept of super efficiency. To comprehensively measure the medical service capacity efficiency of township health centers in various provinces in China, this study adopts the Non-Desirable Output-Super Efficiency SBM model, following Tone's calculation method. The specific formula is as follows:


(1) $$\rho =min\frac{\frac{1}{m}\sum_{i=1}^{m}\frac{\bar{x}}{x_{il}}}{\frac{1}{S_{1}+S_{2}}(\sum_{q=1}^{S_{1}}\frac{y_{q}^{g}}{y_{ql}^{g}}+\sum_{r=1}^{S_{2}}\frac{y_{r}^{g}}{y_{rl}^{g}})}$$

(2) $$\{\begin{array}{c} {\bar{x}}_{i}\geq \sum_{j=1}^{n}\lambda_{j}x_{ij}\\ {\bar{y}}_{q}^{g}\leq \sum_{j=1}^{n}\lambda_{j}y_{qj}^{l}\\ {\bar{y}}_{r}^{d}\leq \sum_{j=1}^{n}\lambda_{j}y_{rj}^{d}\\ {\bar{x}}_{i}\geq x_{io};{\bar{y}}_{q}^{w}\leq y_{qo}^{w};{\bar{y}}_{r}^{b}\geq y_{ro}^{b} \end{array}$$

In this equation, ρ represents the medical service capacity efficiency value of township health centers in various provinces and cities. m is the number of input indicators, S1 is the number of desirable output indicators, S_2_ is the number of non-desirable output indicators, and n is the number of decision-making units. ${\bar{x}}_{i}$, ${ȳ}_{q}^{g}$, ${ȳ}_{r}^{d}$ represent the quantities of input indicators, desirable output indicators, and non-desirable output indicators, respectively, considering slack variables.

### 3.2 Exploratory spatio-temporal data analysis method

Exploratory spatio-temporal data analysis method (25) (ESDA) is a combination of statistics and visualization. It constructs a spatial weight matrix to measure the spatial attribute correlation of neighboring areas and reveal the regional distribution pattern. The correlation is commonly represented by Moran's I index, which includes both global spatial autocorrelation and local spatial autocorrelation.

The formula for Global Moran's I, which represents the global spatial autocorrelation, is as follows:


(3) $$GMI=\frac{n\sum_{i=1}^{n}\sum_{j=1}^{n}W_{ij}(\left(x_{i}-{\bar{x}}_{t}\right)\left(x_{j}-{\bar{x}}_{t}\right))}{\sum_{i=1}^{n}\sum_{j=1}^{n}W_{ij}\sum_{i=1}^{n}{\left(x_{i}-{\bar{x}}_{t}\right)}^{2}}$$

In this equation, n represents the number of decision-making units, ${\bar{x}}_{t}$ t represents the average value of the medical service capacity efficiency for all samples at the grassroots level, *x*_*i*_ and *x*_*j*_ represent the values of neighboring provinces' spatial points, and, *W*_*ij*_ represents the spatial weight. Global Moran's I takes values between −1 and 1. When the Global Moran's I index is between −1 and 0, it indicates a negative correlation and a discrete pattern of medical service capacity efficiency among provinces. When the Global Moran's I index is 0, it indicates no correlation among provinces' medical service capacity efficiency. When the Global Moran's I index is between 0 and 1, it indicates a positive correlation and an agglomeration pattern of medical service capacity efficiency among provinces. The significance of the Global Moran's I index result is verified using the Z-value, for which the formula is as follows:


(4) $$\begin{array}{l} Z\left(GMI\right)=\frac{GMI-E\left(GMI\right)}{\sqrt{Var\left(GMI\right)}} \end{array}$$

In this equation, *E*(*GMI*) represents the theoretical expected value of *GMI* and *Var*(*GMI*) represents the theoretical variance of GMI. When the Z-value passes the significance test, it indicates that the medical service capacity efficiency of township health centers in China exhibits positive spatial correlation.

Local spatial autocorrelation can analyze the correlation characteristics of medical service capacity efficiency among provinces' township health centers. Local spatial autocorrelation decomposes the Global Moran's I index to each province, obtaining the Local Moran's I index. The specific formula is as follows:


(5) $$LMI=\frac{\left(x_{i}-\bar{x})\sum_{j=1}^{n}W_{ij}(x_{j}-\bar{x}\right)}{\sum_{i=1}^{n}{\left(x_{i}-\bar{x}\right)}^{2}}$$

In this equation, the symbols have the same meaning as Equation 4. The Local Moran's I index can partition each province into four quadrants: High-High (HH), Low-High (LH), Low-Low (LL), High-Low (HL). HH indicates that the medical service capacity efficiency of township health centers in the province itself and its neighboring provinces is high; LH indicates that the province itself has low values while its neighboring provinces have high values; LL indicates that the medical service capacity efficiency of township health centers in the province itself and its neighboring provinces is low; HL indicates that the province itself has high values while its neighboring provinces have low values.

Based on the spatial-temporal leap theory proposed by Rey and Janikas (26) and combined with the Moran's I scatter plot, four types of spatial-temporal transitions can be identified. Type I represents self-transition with stable surroundings, Type II represents self-stability with surrounding transitions, Type III represents transitions for both the province itself and its surroundings, and Type IV represents stability for both the province itself and its surroundings. The specific formula for the spatial stability of medical service capacity efficiency among provinces' township health centers is as follows:


(6) $$\begin{array}{l} S=\frac{F_{0,t}}{n} \end{array}$$

In this equation, *F*_0, *t*_ represents the number of sample provinces in period t that exhibit the “self-stability with stable surroundings” state of medical service capacity efficiency in township health centers. *S* value ranges from 0 to 1, where a larger S value indicates stronger spatial stability of medical service capacity efficiency and greater resistance to spatial transitions.

### 3.3 Quantile regression model

Quantile regression (27) is an econometric method proposed by Koenker and Bassett. Its characteristic is that it can split the dependent variable and estimate the trend of the independent variable's impact based on the splitting condition. The estimation results using weighted least absolute deviations are robust, not affected by outliers. The specific formula is as follows:


(7) $$\begin{array}{l} QR^{\theta }=\min X_{i}\sum_{i,LnY_{I}\geq X_{i}QR}^{\theta }\left|LnY_{i}-X_{i}QR\right|+\sum_{i,LnY_{I}X_{i}QR}^{1-\theta }\\ \;\left|LnY_{i}-X_{i}QR\right| \end{array}$$

In this equation, *QR*^θ^ represents the estimated value at different quantiles, with θ representing the quantile. *LnY*_*i*_ represents the observed values at different quantiles, and *X*_*i*_*QR* represents the predicted values at different quantiles. In this study, nine quantiles, namely 0.1, 0.2, 0.3, 0.4, 0.5, 0.6, 0.7, 0.8, and 0.9, are selected for analysis.

## 4 Indicator selection and data source

### 4.1 Indicator selection

This study selected the indicators by drawing on the previous research on the efficiency and driving factors of healthcare service capacity, and by considering data availability and other principles. To eliminate heteroscedasticity, this study logarithmically transformed the driving factors, as shown in Table 1.

**Table 1:** Indicators of efficiency and driving factors.

**Indicator type**	**Indicator name**	**Indicator description**
Input	Number of township health centers	Total number of township health centers in DUM (units)
	Number of healthcare professionals in township health centers	Total number of healthcare technicians in DUM (person)
	Number of beds in township health centers	Total number of beds in DUM (seats)
Expected output	Total number of diagnoses and treatments	Total number of consultations and treatments in township hospitals per year in DUM (number of people)
	Number of discharges	Total number of discharges from township hospitals per year in DUM (people)
Unexpected output	Average length of hospital stay	Average number of days spent hospitalized in township hospitals per year in DUM (day)
Driving factors	Population size	Rural population density (person/km^2^)
	Regional development	Urbanization rate (%)
	Economic development	Per capita disposable income of rural residents (million yuan)
		Per capita gross domestic product (GDP; million yuan)

This study's selected input indicators were the number of township health centers, the number of healthcare professionals in township health centers, and the number of beds in township health centers. Liu et al. (28) explains expected–unexpected output as follows: expected output can be understood as positive output that is determined by internal factors, while unexpected output concerns output that is better in smaller quantities. Researchers have shown that shortening the average length of hospital stay can improve the primary healthcare service capacity (29). Therefore, this study selected the total number of diagnoses and treatments and the number of discharges as the expected output indicators and the average length of hospital stay as the unexpected output indicator.

The driving factors were population size, economic development, policy support, and regional development. To measure population size, this study selected rural population density (lnpd) (30). Population density is a key indicator that reflects regional population distribution, while rural population density eliminates the problem of data untruthfulness caused by differences in the urban–rural population ratio. To measure regional development, this study selected the urbanization rate (lnur) (31). Urbanization refers to the process of the rural population moving to towns or cities as well as the process of transferring production factors to towns or cities. Researchers have shown that accelerated urbanization construction has a positive effect on narrowing the urban–rural gap and promoting urban–rural integration (32). To measure the economic development factors, this study selected rural residents' per capita disposable income (lnpcdi) (33) and per capita gross domestic product (GDP; lnpGDP) (34). Rural residents' disposable income reflects their consumption capacity from a micro perspective, while the per capita GDP can provide a more macro understanding of rural residents' living standards and is an important indicator for measuring macroeconomic development. To measure the policy support factor, this study selected the local governments' healthcare and health expenditure (lnheex) (35). Reasonable and long-term investment in healthcare and healthcare fiscal policies is considered a breakthrough concept for China to deepen its reform of the healthcare and healthcare system. Local governments' healthcare and health expenditure reflects their level of support for the healthcare and healthcare industries.

### 4.2 Data sources

Due to the absence of data for Beijing and Shanghai, this study focuses on the remaining 29 provinces. The relevant data for the years 2009-2021 were collected from the “China Health and Health Statistics Yearbook,” “China Statistical Yearbook,” and “China Rural Statistical Yearbook.” China has been fully implementing the new healthcare reform since 2009, and selecting data with a long time span can also help eliminate the impact of short-term fluctuations on results, making analysis more stable and reliable.

## 5 Empirical result

### 5.1 Efficiency measurement result of healthcare service capacity

(1) Measurement result of the efficiency of the township health centers' healthcare service capacity.

This study used the SBM Run software to measure the efficiency of the healthcare service capacity of township health centers in 29 Chinese provinces from 2009 to 2021. The results are shown in Table 2.

**Table 2:** Efficiency values of the township health centers' healthcare service capacity by province.

**Province**	**2009**	**2010**	**2011**	**2012**	**2013**	**2014**	**2015**	**2016**	**2017**	**2018**	**2019**	**2020**	**2021**	**Mean**	**Rank**
Nationwide	0.667	0.682	0.738	0.731	0.664	0.698	0.738	0.717	0.705	0.688	0.654	0.615	0.591	0.684	
Eastern	0.790	0.774	0.878	0.859	0.787	0.798	0.806	0.807	0.769	0.767	0.730	0.687	0.687	0.780	**2**
Tianjin	0.745	0.582	0.651	0.638	0.565	0.561	0.591	0.550	0.524	0.484	0.337	0.244	0.201	0.513	19
Hebei	0.560	0.557	0.599	0.567	0.519	0.539	0.581	0.624	0.557	0.541	0.461	0.419	0.361	0.530	18
Jiangsu	0.747	0.701	1.031	1.056	1.076	1.088	1.096	1.086	1.133	1.125	1.125	1.126	1.116	1.039	5
Zhejiang	1.221	1.240	1.284	1.300	1.290	1.296	1.303	1.301	1.309	1.317	1.312	1.328	1.330	1.295	1
Fujian	0.723	0.679	0.747	0.695	0.578	0.587	0.600	0.600	0.558	0.591	0.598	0.558	0.569	0.622	15
Shandong	0.680	0.727	1.041	1.080	0.742	0.768	0.747	0.779	0.808	0.792	0.728	0.731	0.777	0.800	13
Guangdong	1.121	1.160	1.088	1.053	1.057	1.048	1.032	1.028	0.814	0.860	0.880	0.820	0.901	0.989	6
Hainan	0.527	0.545	0.582	0.487	0.467	0.498	0.497	0.485	0.451	0.423	0.396	0.266	0.240	0.451	20
Central	0.658	0.707	0.783	0.817	0.691	0.781	0.866	0.876	0.897	0.894	0.828	0.802	0.752	0.796	**1**
Shanxi	0.307	0.301	0.351	0.342	0.307	0.309	0.314	0.331	0.359	0.315	0.278	0.251	0.196	0.305	26
Anhui	0.637	0.620	0.639	0.730	0.681	0.738	0.751	0.724	0.795	0.757	0.735	0.712	0.631	0.704	14
Jiangxi	1.031	1.022	1.058	1.006	0.738	0.798	1.019	1.019	1.030	1.016	0.774	0.680	0.671	0.912	10
Henan	0.788	1.011	1.018	1.018	0.936	1.037	1.066	1.089	1.082	1.116	1.089	1.123	1.134	1.039	4
Hubei	0.587	0.642	0.784	1.009	0.791	1.000	1.022	1.052	1.072	1.083	1.037	1.005	0.848	0.918	9
Hunan	0.599	0.646	0.846	0.797	0.691	0.803	1.023	1.041	1.046	1.078	1.054	1.042	1.033	0.900	11
Westward	0.659	0.679	0.714	0.692	0.652	0.682	0.726	0.666	0.643	0.619	0.609	0.573	0.545	0.651	**3**
Inner Mongolia	0.356	0.358	0.374	0.301	0.309	0.304	0.296	0.326	0.337	0.304	0.217	0.210	0.187	0.298	27
Guangxi	1.105	1.084	1.051	1.065	1.137	1.099	1.069	1.047	1.012	1.006	1.042	1.060	1.072	1.065	3
Chongqing	0.809	0.766	1.016	1.006	0.722	1.040	1.047	1.045	1.051	1.032	1.077	1.061	1.066	0.980	7
Sichuan	1.340	1.203	1.211	1.235	1.137	1.179	1.155	1.149	1.129	1.071	1.116	1.075	1.042	1.157	2
Guizhou	1.132	1.132	1.100	1.096	1.073	1.005	0.600	0.572	0.608	0.680	0.710	0.619	0.661	0.845	12
Yunnan	1.034	1.037	1.025	1.052	1.061	1.061	1.032	1.034	0.783	0.789	0.798	0.846	0.864	0.955	8
Xizang	0.257	0.324	0.305	0.272	0.277	0.259	0.249	0.306	0.302	0.259	0.181	0.131	0.087	0.247	28
Shaanxi	0.354	0.332	0.367	0.369	0.329	0.357	0.386	0.385	0.417	0.429	0.375	0.288	0.277	0.359	25
Gansu	0.124	0.401	0.455	0.455	0.412	0.397	0.423	0.471	0.507	0.505	0.455	0.420	0.362	0.414	21
Qinghai	0.398	0.436	0.539	0.431	0.387	0.375	0.324	0.377	0.361	0.306	0.286	0.268	0.251	0.365	23
Ningxia	0.557	0.592	0.568	0.492	0.454	0.490	1.362	0.484	0.488	0.428	0.402	0.371	0.337	0.540	17
Xinjiang	0.446	0.483	0.551	0.530	0.523	0.617	0.773	0.795	0.718	0.615	0.654	0.532	0.338	0.583	16
Northeast	0.389	0.398	0.371	0.377	0.328	0.330	0.344	0.368	0.395	0.346	0.287	0.215	0.192	0.334	**4**
Liaoning	0.391	0.399	0.452	0.452	0.400	0.411	0.417	0.437	0.473	0.442	0.375	0.281	0.234	0.397	22
Jilin	0.329	0.313	0.305	0.289	0.220	0.223	0.214	0.222	0.255	0.266	0.206	0.154	0.146	0.242	29
Heilongjiang	0.448	0.482	0.355	0.389	0.365	0.356	0.401	0.445	0.459	0.329	0.279	0.209	0.196	0.363	24

Bold text means regional ranking.

(2) Result of the efficiency of the provincial township health centers' healthcare service capacity.

**Figure 1:**
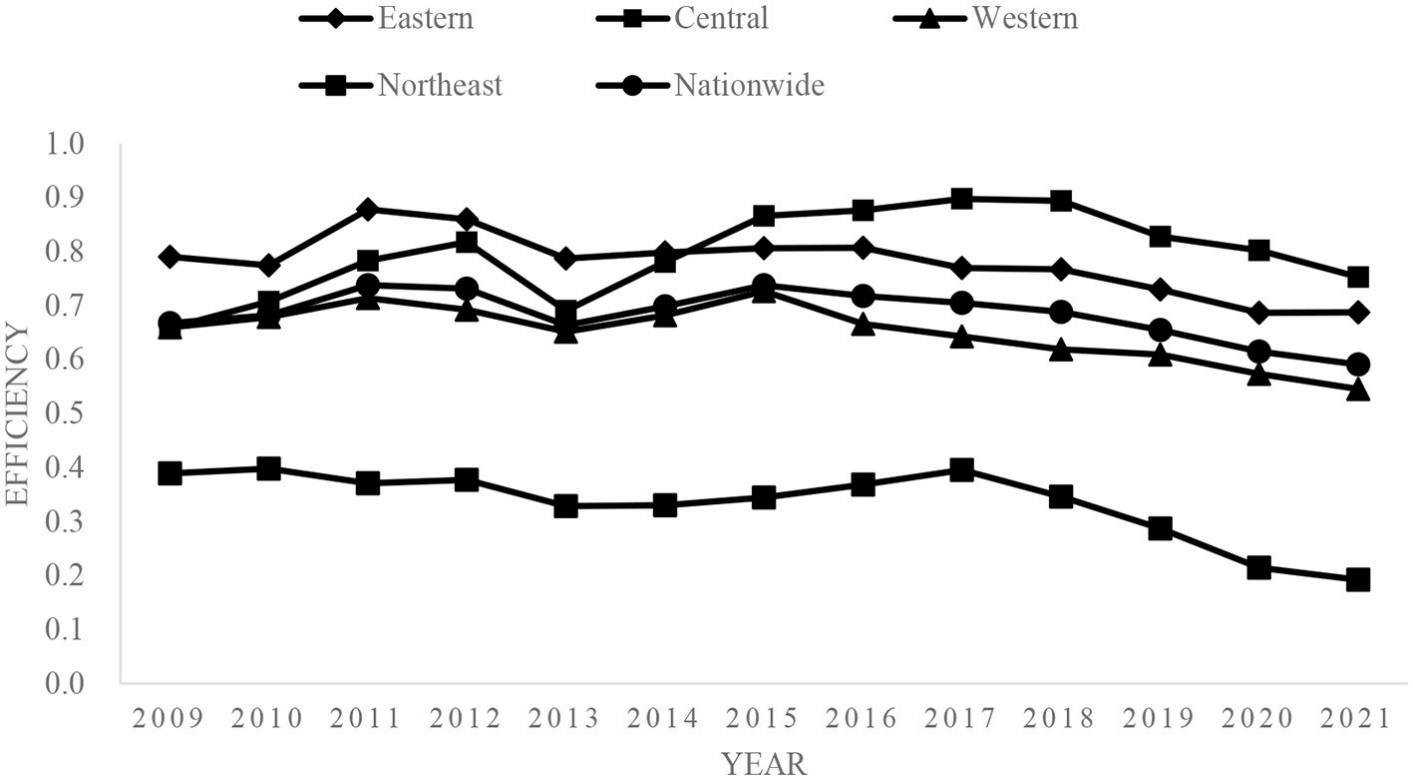
Evolution of provincial medical service capacity and efficiency.

This study used Excel to create an efficiency evolution chart of the provincial healthcare service capacity in the eastern, central, western, and northeastern provincial, as well as nationwide, as shown in Figure 1.

### 5.2 Spatiotemporal evolution result of healthcare service capacity

(1) Empirical result of the global spatial autocorrelation

When the Global Moran's I value is between −1 and 0, the healthcare service capacity of township health centers in each province is negatively correlated and discrete; when the Global Moran's I value is 0, it indicates no correlation; and when the Global Moran's I value is between 0 and 1, it indicates a positive correlation in an agglomerated state. This study used the *z*-value to verify the significance of the Global Moran's I results. When the *z*-value passed the significance test, the healthcare service capacity of township health centers had a positive spatial correlation. This study used Stata 17.0 software to obtain the Global Moran's I values for the efficiency of the township health centers' healthcare service capacity in 29 Chinese provinces for 2009–2021. The results are shown in Table 3.

**Table 3:** Global Moran's I values, 2009–2021.

**Year**	***I***	***E*(*I*)**	***SD*(*I*)**	***Z***	***P***
2009	0.348	−0.036	0.105	3.670	0.000
2010	0.386	−0.036	0.105	4.012	0.000
2011	0.413	−0.036	0.106	4.241	0.000
2012	0.400	−0.036	0.106	4.115	0.000
2013	0.311	−0.036	0.105	3.293	0.001
2014	0.327	−0.036	0.106	3.433	0.001
2015	0.272	−0.036	0.106	2.916	0.004
2016	0.355	−0.036	0.106	3.695	0.000
2017	0.349	−0.036	0.105	3.643	0.000
2018	0.390	−0.036	0.106	4.031	0.000
2019	0.355	−0.036	0.106	3.697	0.000
2020	0.355	−0.036	0.106	3.699	0.000
2021	0.379	−0.036	0.106	3.928	0.000

I, Moran's I value; E(I), Expected value; SD(I), Standard deviation; Z, Z value; P, P value.

(2) Empirical result of the local spatial autocorrelation

Local spatial autocorrelation can be used to analyze the correlation characteristics of the healthcare service capacity of township health centers in each province. Local spatial autocorrelation decomposes the Global Moran's I of each province to obtain the Local Moran's I. The Local Moran's I divides each province into four quadrants: high-high (HH), low-high (LH), low-low (LL), and high-low (HL). HH represents a high value of healthcare service capacity of township health centers in the province and neighboring provinces, LH represents a low value of healthcare service capacity in the province itself and a high value in neighboring provinces, LL represents a low value of healthcare service capacity in the province and neighboring provinces, and HL represents a high value of healthcare service capacity in the province itself and a low value in the neighboring provinces. Following Rey and Janikas (26) spatiotemporal leap theory combined with the Moran's I scatter plot, this study derived four spatiotemporal leap transition types: Type I, self-leaping and surrounding stability; Type II, self-stability and surrounding leaping; Type III, self-leaping and surrounding leaping; and Type IV, self-stability and surrounding stability. To better characterize the spatial agglomeration trend of each province, this study selected the Local Moran's I values of each province in 2009, 2015, and 2021, according to the efficiency trend changes. The results are shown in Table 4.

**Table 4:** Quadrant distribution of the Local Moran's I, 2009–2021.

**Year**	**First quadrant (HH)**	**Second quadrant (LH)**	**Third quadrant (LL)**	**Fourth quadrant (HL)**
2009	Jiangsu/Zhejiang/Fujian/ Shandong/Guangdong/Jiangxi/ Henan/Guangxi/Chongqing/ Sichuan/ Yunnan/Guizhou	Hainan/Anhui/ Hubei/Hunan/ Qinghai/Xizang	Hebei/Shanxi/Inner Mongolia/Shaanxi/ Gansu/Ningxia/ Xinjiang/Liaoning/ Jilin/Heilongjiang	Tianjin
2015	Jiangsu/Zhejiang/ Shandong/Guangdong/ Jiangxi/Henan/Anhui/ Hubei/ Hunan/Guangxi/ Chongqing/Yunnan	Hainan/Fujian/ Qinghai/Xizang/ Guizhou	Hebei/Tianjin/Inner Mongolia/Shanxi/ Shaanxi/Gansu/ Liaoning/ Jilin/Heilongjiang	Sichuan/Ningxia/Xinjiang
2021	Jiangsu/Zhejiang/ Shandong/Guangdong/ Jiangxi/Henan/Anhui/ Hubei/Hunan/Guangxi/ Chongqing/Yunnan/ Guizhou	Hainan/Fujian/ Qinghai/Xizang	Hebei/Tianjin/ Inner Mongolia/ Shanxi/Shaanxi/ Gansu/Ningxia/ Xinjiang/Liaoning/ Jilin/Heilongjiang	Sichuan

(3) Spatiotemporal leap result

This study analyzed the spatiotemporal leap pattern of the provincial township health centers' healthcare service capacity using the empirical local spatial autocorrelation results. The leap type results are shown in Table 5.

**Table 5:** Spatiotemporal leap types of the provincial township health centers' healthcare service capacity.

**Leap type**	**2009–2015**	**2016–2021**
Type I	HH-LH: Fujian/Guizhou LH-HH: Hunan/Hubei/Anhui HL-LL: Tianjin LL-HL: Ningxia/Xinjiang	LH-HH: Guizhou HL-LL: Ningxia/Xinjiang
Type II	HH-HL: Sichuan	-
Type III	-	-
Type IV	HH-HH: Jiangsu/Shandong/Chongqing/Henan/ Yunnan/Guangxi/Jiangxi/Guangdong/ Zhejiang LL-LL: Hebei/Shanxi/Shaanxi/Gansu/Liaoning/Jilin/Inner Mongolia/Heilongjiang	HH-HH: Jiangsu/Yunnan/Jiangxi/Guangxi/ Chongqing/Hunan/Guangdong/ Zhejiang/Henan/Hubei/Anhui/Shandong LH-LH: Fujian/Hainan/Qinghai/Xizang LL-LL: Gansu/Shanxi/Shaanxi/Tianjin/ Hebei/Jilin/Inner Mongolia/Liaoning/ Heilongjiang HL-H: Sichuan

### 5.3 Result of the driving factors of healthcare service capacity

(1) Quantile regression result

This study used Stata 17.0 software to calculate the quantile regression values of the driving factors of healthcare service capacity. The results are shown in Table 6.

**Table 6:** Results of quantile regression and ordinary least squares regression for the drivers of healthcare service capacity.

	**con_s**	**lnpd**	**lnur**	**lnpcdi**	**lnpGDP**	**lnheex**
Ordinary least squares	−2.394^***^	0.129^***^	−0.622^***^	−0.448^**^	0.136^***^	0.685^***^
0.1	−3.336^***^	0.130^***^	−0.868^***^	−0.040	0.113^**^	0.455
0.2	−2.382^***^	0.097^***^	−0.585^***^	−0.765	0.138^***^	0.941^***^
0.3	−1.984^***^	0.094^***^	−0.506^***^	−0.796^**^	0.144^***^	0.902^***^
0.4	−2.037^***^	0.117^***^	−0.495^***^	−0.914^***^	0.109^***^	1.05^***^
0.5	−1.884^**^	0.124^***^	−0.490^***^	−1.097^***^	0.133^***^	1.156^***^
0.6	−1.312	0.126^***^	−0.470^***^	−1.218^***^	0.196^***^	1.083^***^
0.7	−0.588^***^	0.126^***^	−0.434^***^	−0.765	0.222^***^	0.526
0.8	0.241^***^	0.126^***^	−0.262^*^	−0.564^*^	0.204^***^	0.231
0.9	−0.210	0.159^***^	−0.190	0.726	0.050	−0.597

^***^p<*0.01;* ^**^p<*0.05;* ^*^p<*0.1; con_s: constant term*.

(2) Nested spatiotemporal leaps and quantile regression result

This study followed the quantile regression steps of Zhang et al. (36) and Ma et al. (37). This study used the 0.1–0.5 quantile as the low quantile response and the 0.6–0.9 quantile as the high quantile response. Based on the sign differences, the quantiles were divided into four pattern types: low quantile response and significant positive effect: low quantile-driven pattern; low quantile response and significant negative effect: low quantile-constrained pattern; high quantile response and significant positive effect: high quantile-driven pattern; and high quantile response and significant negative effect: high quantile-constrained pattern. The spatiotemporal leap results for 2009–2021 were nested within the four pattern types. The results are shown in Table 7.

**Table 7:** Nested results of spatiotemporal leaps and quantile regression.

**Quantile response mode**	**Driving type**	**Leap province**
Low quantile driving	Parallel development	LH-HH (Anhui/Hubei/ Hunan) LL-HH (0)
	Reverse development	LH-HL (0) LL-HL (0)
Low quantile constraining	Mutual constraint	LH-LH (Hainan/Qinghai/ Xizang) LL-LL (Hebei/Shanxi/ Shaanxi/Inner Mongolia/ Gansu/Ningxia/ Xinjiang/ Liaoning/Jilin/Heilongjiang)
	Reverse development	LH-LL (0) LL-LH (0)
High quantile driving	Parallel development	HH-HH (Jiangsu/Zhejiang/ Shandong/ Guangdong/Jiangxi/ Henan/Guangxi/ Chongqing/Yunnan/ Guizhou) HL-HL (0)
	Reverse development	HH-HL (Sichuan) HL-HH (0)
High quantile constraining	Mutual constraint	HH-LH (Fujian) HL-LL (Tianjin)
	Reverse development	HH-LL (0) HL-LH (0)

## 6 Discussion

### 6.1 Efficiency analysis of medical service capacity

Regarding the changes in efficiency, the efficiency results for the healthcare service capacity of township health centers can be divided into two stages. The first stage is 2009–2015, during which the overall efficiency value shows an upward trend. The number of provinces with efficiency values greater than 1 increased from 7 in 2009 to 12 in 2015, with small fluctuations observed between 2011 and 2015. The policy research found that at this stage, healthcare service items were reformed because of township health centers' limited healthcare resources (38). Basic public health service items were mainly undertaken by township health centers, which weakened their healthcare service capacity to some extent. Subsequently, the Chinese government stimulated the development of township health centers through financial investment and other means. The second stage is 2016–2021, during which a downward trend in efficiency emerges. The number of provinces with efficiency values greater than 1 decreased from 11 in 2016 to 7 in 2021. During this period, the Chinese government implemented strict budget mechanisms from the administrative and economic perspectives, and township health centers gradually developed into nonprofit healthcare institutions. 2016 is the first year of the “13th Five-Year Plan”. The Notice on Deepening the Key Tasks of the Reform of the Medical and Health System in 2016 triggered by The General Office of the State Council mentioned comprehensively deepening the reform of public hospitals. Put forward specific measures for the tasks of government responsibility, hospital management system and personnel system. From an economic point of view, the policy of canceling drug addiction has an impact on the income of township health centers, which means that township health centers are gradually developing into non-profit medical institutions. However, this approach also led to a reduction in the township health centers' profits (39). At the same time, since 2016, the number of township health institutions in most provinces has decreased year by year, which is another reason for the change in efficiency value.

The provinces can be divided into five categories based on their average efficiency values. The first category is Zhejiang, Sichuan, Guangxi, Henan, and Jiangsu, with efficiency values in the range [1, ∞). The second category is Guangdong, Chongqing, Yunnan, Hubei, Jiangxi, and Hunan, with efficiency values in the range [0.9, 1), mainly in the central region. The third category is Guizhou, Shandong, Anhui, Fujian, Xinjiang, Ningxia, Hebei, and Tianjin, with efficiency values in the range [0.5, 0.9). The fourth category is Hainan, Gansu, Liaoning, Qinghai, Heilongjiang, Shanxi, and Shaanxi, with efficiency values in the range [0.3, 0.5). The fifth category is Inner Mongolia, Tibet, and Jilin, with efficiency values in the range [0, 0.3). The proportion of hierarchical diagnoses and treatment pilot projects in cities in the first category is relatively large; 11 prefecture-level cities in Zhejiang and 21 administrative units in Sichuan have become pilot cities for hierarchical diagnoses and treatments, and the efficiency values for both provinces remain above 1. This result is consistent with that of Gao et al. (40). Regarding the average annual growth rate, the rates for Gansu, Hunan, Jiangsu, Hubei, Henan, Chongqing, Shandong, and Zhejiang are positive, while those for the remaining 22 provinces are negative, which relates to the health funding allocated by local governments to township health centers.

Overall, the differences among the four major regions are quite obvious, with the general trend showing the order central > eastern > western > northeastern. This trend is consistent with Pei's results (41). Zhang (42) found that both the actual reimbursement rate for hospitalization and the number of residents who were hospitalized in township health centers were highest in the central region. Regarding the efficiency trend of healthcare service capacity in township health centers, the eastern region was the highest from 2009 to 2014 but was overtaken by the central region in 2015. The efficiency value began to decline in 2011 and showed a clear upward trend from 2021. In the central region, the efficiency value showed an upward trend for 2009–2012, a significant downward trend during 2012–2013, a rebound from 2013 to 2018, and a slight downward trend for 2018–2021. In the western region, the efficiency value shows a slight fluctuation trend from 2009 to 2013, displaying a reverse-U-shape, and shows an upward trend from 2013 to 2015, but continuously declined from 2015 to 2021. In the northeastern region, the efficiency value remained stable from 2009 to 2017 but showed a significant downward trend from 2017 to 2021. Thus, the efficiency measurements of the healthcare service capacity of township health centers in the four regions declined at different times. In the interviews conducted by the research team in 2018 in Shandong, Zhejiang, Gansu, and other places, the respondents revealed that rural residents' increasing health demands could not be met by the healthcare resources and technical skills held by township health centers. For example, the township health centers could only perform simple auxiliary examinations, such as X-rays and ultrasounds, while routine auxiliary examinations, such as CT scans, could only be performed in county-level hospitals or higher. Moreover, regarding the per capita healthcare expenses, the proportion of personal expenditure has continued to rise, which has led rural residents to be more inclined to choose county- or city-level hospitals for healthcare treatment.

In conclusion, the efficiency of healthcare service capacity in township health centers is better in provinces with a higher proportion of hierarchical diagnoses and treatment pilot projects in cities. In terms of regional distribution, the efficiency of healthcare service capacity generally shows the order of “central > eastern > western > northeastern,” and the efficiency of the healthcare service capacity in township health centers in the four regions has declined at different times. China should expand the pilot program of tiered diagnosis and treatment in cities. Provinces should fully utilize administrative measures and economic levers to form an incentive mechanism for preliminary diagnoses at the grassroots level, two-way referrals, and multichannel guidance; promote the expansion and sinking of high-quality healthcare resources; alleviate the structural imbalance of healthcare resource allocation; and improve the grassroots healthcare service capacity by accelerating the construction of national and regional healthcare centers, expanding the construction of healthcare consortia, and improving relevant policies for tiered diagnoses and treatments.

### 6.2 Space-time evolution analysis of medical service ability

The Global Moran's I values for the efficiency of the healthcare service capacity in each year are positive and pass the test at different levels of significance, indicating that the efficiency of the healthcare service capacity has a significant spatial correlation with the spatiotemporal distribution. The Global Moran's I results can be divided into three stages. The first stage is 2009–2011, during which the efficiency of the healthcare service capacity exhibits a continuous upward trend. The second stage is 2011–2015, during which the efficiency of the healthcare service capacity shows an overall downward trend, except for a slight increase from 2013 to 2014. The third stage is 2015–2021, during which the efficiency of the healthcare service capacity shows a fluctuating upward trend. The efficiency trend changes in the healthcare service capacity are increasingly influenced by neighboring provinces, and there is both competition and cooperation among the provinces. Accordingly, China has implemented two assistance mechanisms: vertical and horizontal. The vertical assistance mechanism refers to the support between healthcare consortia, whereas the horizontal assistance mechanism refers to the support between healthcare centers at the same level. Township health centers have also developed network-style layouts.

From an overall perspective, over the period 2009–2021, the efficiency of the healthcare service capacity in each province is mainly distributed in the HH and LL quadrants, indicating a strong spatial clustering pattern with predominantly positive spatial autocorrelation. Regarding the provincial changes, in 2009, 12 provinces are in the HH quadrant and 10 are in the LL quadrant. In 2015, 12 provinces are in the HH quadrant and 9 provinces are in the LL quadrant. In 2021, 13 provinces are in the HH quadrant and 11 are in the LL quadrant. The provincial trend further confirms that the spatial distribution of the healthcare service capacity has a strong positive correlation, with relatively few provinces in the LH and HL quadrants, thus exhibiting a “high agglomeration, low differentiation” spatial distribution.

Regarding the regional trend, in 2009, the HH agglomeration pattern is mainly distributed in the eastern region. The relevant GDP data shows that the eastern region's economic development is faster than that of other regions, and that more healthcare resources can be supplied to a certain extent, which is conducive to improving the healthcare service capacity of township health clinics. The LL agglomeration pattern is mainly distributed in the western and northeastern regions, where there are relatively more rural populations and a greater consumption of primary healthcare resources. The central region mainly exhibits an LH agglomeration pattern, indicating that the positive influence of neighboring provinces increases the efficiency of the healthcare service capacity. In 2015, the HH agglomeration pattern starts to spread to the central region, as backup advantages [Human, Financial, and Material resources (43)] are utilized and the corresponding investments are increased, resulting in faster efficiency improvement. The LL agglomeration pattern mainly occurs in the western and northeastern regions. In 2021, the differences are smaller than in 2015, and the western and northeastern regions mainly exhibit the LL agglomeration pattern, indicating that China should focus on these regions.

The provinces mostly show the Type IV leap pattern, while no province shows the Type III pattern, indicating that the overall spatiotemporal evolution of each province is stable, and there are no features of extreme instability. Specifically, from 2009 to 2015, Fujian and Guizhou have a leap type of HH-LH. Fujian is a coastal province with rapid economic development and increasing demand for healthcare services, whereas Guizhou is affected by its neighboring provinces, leading to a decrease in the efficiency of healthcare service capacity. Hunan, Hubei, and Anhui were positively influenced by their neighboring provinces, leaping from LH to HH in terms of healthcare service capacity, while Tianjin's healthcare service capacity declined due to its negative neighboring influence, leaping from HL to LL. Ningxia and Xinjiang have a leap type of LL-HL, which may relate to the greater supply of healthcare resources due to economic development. Sichuan shows a positive local influence, resulting in a decrease in the healthcare service capacity of its neighboring provinces, and leaps from HH to HL. From 2016 to 2021, Guizhou showed a leap type of LH-HH due to a positive neighboring influence, leading to an increase in its healthcare service capacity, whereas Ningxia and Xinjiang are negatively influenced by neighboring provinces, leading to a decrease in their healthcare service capacity, with both provinces having a leap type of HL-LL.

In conclusion, the global spatial autocorrelation results indicate that the efficiency of healthcare service capacity in township health centers in each province has a significant spatial correlation with its spatiotemporal distribution, gradually forming a grid layout. From the local spatial autocorrelation results, the efficiency of healthcare service capacity mainly reveals the HH and LL distribution patterns, showing the characteristics of “high agglomeration, low differentiation.” From the spatiotemporal leap results, the provinces mostly show the Type IV leap pattern, while no province shows the Type III pattern. China should establish national collaborative development models. Provincial governments should adopt a model of deep cooperation; build a nationwide healthcare information system that is interconnected and shared; and improve support mechanisms, such as multi-point practice and counterpart support regarding systems and technologies. They should also promote the excellent experiences and practices of township health centers that have good development status, provide a reference for township health centers northeastern, and promote the innovative development of township health centers based on their previous experience and actual local conditions, so as to effectively improve the grassroots healthcare service capacity.

### 6.3 Analysis of influencing factors of medical service ability

Lnpd was significantly tested at each quantile (0.1–0.9); the results showed a promoting effect on the efficiency of healthcare service capacity. This is because areas with denser rural populations require more healthcare resources, which stimulates township health centers' healthcare service capacity to some extent. Moreover, in reality, areas with high rural population density tend to receive a certain proportion of increased government investment, which is consistent with the results of Chen and Han (44). Lnur shows a statistical difference in all quantiles except the 0.9 quantile, indicating an inhibitory effect. Although urbanization drives rural development, it also leads to the flow of rural populations to urban areas, resulting in some rural areas becoming empty-nest villages. This causes both a healthcare concentration and a decline in the efficiency of the healthcare service capacity of township health centers; this result is consistent with that of Wang and Han (45). Lnpcdi is significantly tested in the low (0.3–0.5) and high (0.6, 0.8) quantiles; the coefficients were negative, indicating that the per capita disposable income of rural residents has an inhibitory effect on the efficiency of healthcare service capacity. This may be because as rural residents' income increases, their demand for healthcare becomes more urgent. They believe that urban hospitals have more abundant healthcare resources and are better able to meet their health needs, resulting in a decline in the efficiency of healthcare service capacity of township health centers, which is consistent with the results of Yang and Lü (15). LnpGDP is not significant in the 0.9 quantile, the other quantiles show statistical differences and exhibit a promoting effect. Generally, the higher a province's economic development level, the more funds the government can disperse for grassroots healthcare services. Jin et al.'s (46) results also indicate that in the New Healthcare Reform stage, regions with better economic development have smaller differences in the supply of basic healthcare resources and decreases in the differences in per capita GDP.Lnheex is significant in the low quantile (0.2–0.5) and high quantile (0.6) tests, and the coefficients are positive, indicating that it has a promoting effect on the efficiency of healthcare service capacity of township health centers. Increasing local governments' healthcare and health expenditure positively affects reducing residents' healthcare expense burden. According to the studies, from the supply perspective, local governments' healthcare and health expenditure is mainly used for healthcare infrastructure and healthcare staff salaries. Having fully-equipped healthcare infrastructure in township health centers can effectively improve rural residents' healthcare needs, while an increase in healthcare staff salaries can effectively improve the quality of healthcare services provided. However, from the regression results, local governments' healthcare and health expenditure reaches saturation after a certain increase, which is similar to the result of Wang and Hu (47).

In the low quantile-driving pattern, lnpd, lnpGDP, and lnheex are the most significant factors that affect the spatiotemporal efficiency leap of the healthcare service capacity of township health centers. They have a significant promoting effect on the development of the healthcare service capacity in Anhui, Hubei, and Hunan. In the low quantile-constraining pattern, lnur and lnpcdi constrain the spatiotemporal leap in the healthcare service capacity of township health centers. In this mode, the rapid promotion of urbanization and improvement of per capita disposable income is conducive to enhancing the healthcare service capacity of township health centers in Hebei, Shanxi, and Inner Mongolia. In the high quantile-driving pattern, lnpd, lnpGDP, and lnheex have a significant promoting effect on the spatiotemporal leap in the healthcare service capacity of township health centers. This pattern is mainly observed in the eastern region, indicating that the economic development characteristics of provinces such as Jiangsu, Zhejiang, and Shandong promote the enhancement of the healthcare service capacity of township health centers. Local governments' fiscal health expenditure passes the significance test in the high quantile response (0.6), indicating that it will have a significant promoting effect when it reaches a certain level. In the high quantile-constraining pattern, lnur and lnpcdi are the main factors that constrain the spatiotemporal leap in the healthcare service capacity of township health centers. With the continuous acceleration of urbanization and improvement of residents' living standards, provinces such as Fujian and Tianjin constrain the spatiotemporal leap in the healthcare service capacity of township health centers. Judging from the characteristics of these two provinces, the urbanization level is relatively high, and the rural residents are gradually moving to the cities. Therefore, the development of township health centers will inevitably be affected to some extent, which is consistent with the actual situation.

In conclusion, the quantile regression results show that rural population density and per capita GDP significantly promote the improvement of the healthcare service capacity, and local governments' healthcare and health expenditure has a promoting effect on the healthcare service capacity of township health centers in certain quantiles. The urbanization rate and per capita disposable income inhibit the improvement of the healthcare service capacity of township health centers at certain quantiles. From the nested results of the spatiotemporal leaps and quantile regression, rural population density and local governments' healthcare and health expenditure promote the healthcare service capacity of township health centers in Anhui, Hubei, Hunan, and other provinces. Per capita GDP promotes the healthcare service capacity of township health centers in Jiangsu, Zhejiang, Shandong, Guangdong, and other provinces, whereas the level of urbanization restricts the leap in healthcare service capacity of township health centers in Tianjin and Fujian. China should guide each province in formulating implementation plans that meet their actual needs. Provinces such as Jiangsu, Zhejiang, Shandong, and Guangdong can adopt differentiated measures to improve their healthcare service capacity, such as by increasing local per capita income and increasing investment in healthcare and healthcare. Doing so will also have a radial effect on the neighboring provinces. Tianjin and Fujian Provinces can improve their grassroots healthcare service capacity by adjusting for population size and other measures.

## 7 Conclusion

Through the analysis of the medical and health service capacity of township hospitals in China, the efficiency of graded diagnosis and treatment pilot areas is higher. But in terms of time, efficiency has declined in all regions.The characteristics of “high aggregation, low differentiation” indicate that the ability of medical and health services will be affected by neighboring provinces. In addition, due to the differences in population, economy and other factors, the shortcomings of the medical service capacity of township health centers in various provinces are also different. The state should expand the trial of graded diagnosis and treatment in cities, establish national collaborative development models, and guide each province in formulating implementation plans that meet their actual needs.

## 8 Innovation and deficiency

The purpose of this paper is to accurately grasp the regional differences in the development of medical service capacity of township health centers in China, and to improve the medical service capacity of township health centers is a key issue for the government to actively promote the reform of the medical and health system. This paper comprehensively uses the non-expected output SBM model, exploratory spatiotemporal data analysis method and quantile regression to study the efficiency analysis, spatiotemporal evolution characteristics and driving factors of the medical service capacity of township health centers in China, which can fully reflect the quality of the medical service capacity of township health centers in China, and has a guiding role in regulating the medical service of township health centers. It can also be used for reference in other areas.

Although this study systematically and empirically assessed the efficiency, spatiotemporal evolution characteristics, and driving factors of the healthcare service capacity of township health centers in Chinese provinces, the data on the township health centers at the provincial level only allowed for the characterization of strategies for improving the healthcare service capacity of township health centers from a top-down perspective. However, there may be significant differences in the specific situation of each township in each province, which could lead to deficiencies in the institutional construction and bottom-up logic. Therefore, future research should collect and study the micro data on each township's health center.

Further, the driving factors selected herein were only obtained through a literature review, which may give rise to endogeneity issues owing to the lack of identification of critical factors in practical processes. Therefore, future research should conduct in-depth interviews to further explore the factors that may affect the healthcare service capacity of township health centers.

## Data availability statement

The datasets presented in this study can be found in online repositories. The names of the repository/repositories and accession number(s) can be found at: https://data.stats.gov.cn/easyquery.htm?cn=C01 and http://www.nhc.gov.cn/mohwsbwstjxxzx/tjzxtjcbw/tjsj_list.shtml.

## Author contributions

Among the contributing authors of this study, HC was engaged in study design, data processing, and analysis and paper writing. LZ was engaged in study design and involved in data analysis. JY actively participated in study design and field survey. All authors of this article have read and approved the final manuscript.
